# Supplementary material for: Early functional connectivity alterations in contralesional motor networks influence outcome after severe stroke: a preliminary analysis
Source: Sci Rep. 2023 Jul 7;13:11010. doi: 10.1038/s41598-023-38066-0 (PMC10328915; doi:10.1038/s41598-023-38066-0)
Supplement: Supplementary file 1 — Supplementary Information. [file 41598_2023_38066_MOESM1_ESM.pdf]

Early functional connectivity alterations in contralesional motor networks influence outcome after severe stroke– a preliminary analysis

Hanna Braaß*#, Lily Gutgesell*, Winifried Backhaus, Focko L. Higgen, Fanny Quandt, Chi-un Choe, Christian Gerloff and Robert Schulz

Department of Neurology, University Medical Center Hamburg Eppendorf, Martinistr. 52, 20246 Hamburg, Germany

* Contributed equally

Corresponding author:
Hanna Braaß
University Medical Center Hamburg-Eppendorf
Martinistraße 52, 20246 Hamburg, Germany
Phone: +49-40-7410-55573
Email: h.braass@uke.de

Supplementary Material

ID	Age	Sex	Side / Dom	TL / MT (TICI)	LVO	LV (ml)	Days C/I	NIHSS		UEFM		MRS		BI	
								Acute	$T_{2/3}$	Acute	$T_{2/3}$	Acute	$T_{2/3}$	Acute	$T_{2/3}$
1	78	female	left / d	no / no	none	33.6	7/7	10	3	8	31	4	3	35	80
2	63	male	left / d	no / no	M1	55.8	3/3	13	1	5	36	4	1	40	100
3	73	female	left / d	yes / yes (2B)	M1	14.4	5/5	9	3	48	65	4	3	20	100
4	73	female	right / n	yes / yes (2A)	M1	27.6	5/5	5	2	49	62	4	1	25	80
5	79	female	right / n	yes / yes (2B)	M2 / A1	120.4	5/6	8	2*	15	51*	5	4	10	85*
6	89	female	right / n	yes / no	none	2.6	4/4	7	3	5	39	5	3	40	55
7	71	female	right / n	yes / yes (2A)	ACI/M1/A1	38.4	8/8	9	-	4	47*	5	3*	10	70*
8	76	male	right / n	yes / yes (3)	M1/A2	101.0	5/6	11	-	4	-	5	3†	10	-
9	78	male	right / n	yes / yes (3)	M2	178.1	4/4	17	3	5	15	5	4	10	65
10	85	female	right / n	no / yes (2B)	M1	33.5	5/5	15	14	2	4	5	5	0	0
11	78	male	left / d	no / no	M1	58.1	10/15	17	-	3	-	5	5*,†	10	15*,†
12	74	male	left / d	yes / yes (2B)	M1	303.3	10/10	24	-	5	4*	5	5	5	5
13	69	male	left / d	yes / yes (2A)	M1	98.4	7/7	18	-	0	-	5	-	0	-
14	77	female	right / n	yes / no	M1	286.7	7/7	11	10*	4	4*	4	4*	20	45*
15	67	female	right / n	yes / no	none	7.4	8/7	11	7	6	5*	4	3	30	95
16	58	female	left / d	no / no	ACI/M1	58.4	13/13	23	-	0	-	5	-	5	-
17	80	female	left / d	no / no	M1	20.5	12/12	11	15*	-	4*	5	4*	0	30*
18	83	female	left / d	yes / yes (3)	Carotis-T	21.6	9/9	10	-	6	-	5	-	20	-
19	80	male	right / n	yes / yes (0)	M1	108.4	7/7	16	-	6	-	5	6†	10	-
**Stroke**	73.8 (5.8)	7 male	10 right / 9 d	13 TL / 11 MT	mode: M1	82.5 (87.6)	mode: 5/7	12.89 (5.17)	5.7 (5.0)	9.7 (14.5)	30.3 (23.3)	4.7 (0.5)	3.6 (1.4)	15.8 (13.0)	58.9 (34.8)
**Controls**	75.3 (7.5)	7 male	-	-	-	-		-	-	-	-	-	-	-	-

Supp. Tab. 1| Patient characteristics at baseline and functional improvement over time.

Baseline characteristics of all patients individually and averaged per group, for patients and controls. Mean values and standard deviation in brackets are given. Side lesion side. Dom Hemispherical dominance with d dominant hemisphere, n non-dominant hemisphere affected by the lesion. TL thrombolysis, MT mechanical thrombectomy, TICI: thrombolysis in cerebral infarction grading system, partial perfusion of the treated vessel is reached in grade 2B (mode). LVO large vessel occlusion. LV lesion volume. M1/M2/A1/ACI indicate the cerebral vessels occluded. Days C/I Exam time point of clinical examination (C) and imaging (I) in days after stroke. Recovery and initial scoring of four scores: NIHSS National Institute of Health Stroke Scale, UEFM Upper Extremity Fugl Meyer Assessment, MRS modified Rankin Scale, BI Barthel-Index. Time point of data collection in the acute (day 3-14) or late sub-acute stage (**$T_{2/3}$**), either three or six months (depicted by *) after stroke. † indicate follow-up values collected via phone.

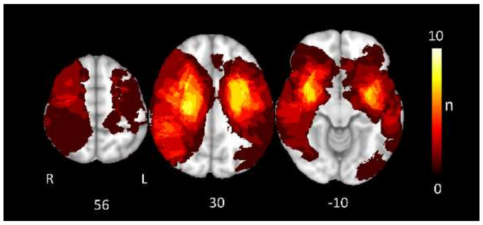

Supp. Fig. 1 | Frequency map of the stroke lesions in the right and left hemisphere
Displayed are frequency maps of the stroke lesions in the right and left hemisphere, overlaying a T1-weighted template in MNI space (z-coordinates below each slice). The color intensity indicated the number of subjects of whom lesion voxels lay within the colored region.
